# COVID-19 and Female Fertility: An Observational Prospective Multicenter Cohort Study: Upholding Reproductive Rights in Emergency Circumstances

**DOI:** 10.3390/diagnostics14192118

**Published:** 2024-09-24

**Authors:** Giuseppe Gullo, Alessandra Lopez, Carla Loreto, Gaspare Cucinella, Marco La Verde, Alessandra Andrisani, Sofia Burgio, Raffaela Carotenuto, Silvia Ganduscio, Giovanni Baglio, Valentina Billone, Antonio Perino, Pasquale De Franciscis, Susanna Marinelli

**Affiliations:** 1 Department of Obstetrics and Gynecology, IVF Unit, AOOR Villa Sofia Cervello, University of Palermo, 90146 Palermo, Italy; alessandralopez91@gmail.com (A.L.); gaspare.cucinella@unipa.it (G.C.); gandusciosilvia@gmail.com (S.G.); valentina.billone@gmail.com (V.B.); antonio.perino@unipa.it (A.P.); 2 Department of Woman, Child and General and Specialized Surgery, University of Campania “Luigi Vanvitelli”, 80121 Naples, Italy; loretocarla@virgilio.it (C.L.); marco.laverde@unicampania.it (M.L.V.); raffaela.carotenuto95@gmail.com (R.C.); pasquale.defranciscis@unicampania.it (P.D.F.); 3 Unit of Gynecology and Obstetrics, Department of Women’s and Children’s Health, University of Padua, 35128 Padua, Italy; alessandra.andrisani@unipd.it; 4 Italian National Agency for Regional Healthcare Services, 00187 Roma, Italy; baglio.giovanni@yahoo.it; 5 School of Law, Polytechnic University of Marche, 60121 Ancona, Italy; susanna.marinelli@tiscali.it

**Keywords:** SARS-CoV-2 pandemic, female fertility, anti-Müllerian hormone (AMH), antral follicle count (AFC), ART access

## Abstract

Objectives: Currently available research data points to COVID-19-related multi-organ system damage. This study aims to evaluate the impact of SARS-CoV-2 on the reproductive health, that is, plasma levels of FSH, LH, estradiol, AMH, and antral follicular count, of women undergoing level II ART techniques. Methods: This is a multicenter, prospective, and observational study by the reproductive medicine centers of Palermo’s Ospedali Riuniti Villa Sofia-Cervello Hospital and Vanvitelli University. From September 2022 to March 2024, 203 patients aged 24–43 were enrolled, all with diagnosed infertility and a history of SARS-CoV-2 infection. Symptomatic women, patients testing positive for HIV or other liver viruses, and patients with a history of ovarian cancer or who had taken gonadotoxic drugs were excluded. Plasma measurements of FSH, LH, estradiol, AMH, and antral follicular count were performed before and after infection. Results: The analysis accounting for the concentration of anti-Müllerian hormone (AMH) before and after COVID-19 infection shows an average concentration decrease from 1.33 ng/mL before SARS-CoV-2 infection to 0.97 ng/mL after infection. Average decrease after infection was −27.4%; average reduction of 1 follicle (95% CI: from −0.74 to −1.33) was reported following SARS-CoV-2 infection. Levels of E2 before and after SARS-CoV-2 infection did not vary significantly. Average FSH and LH levels before and after SARS-CoV-2 infection pointed to an increase. Conclusions: SARS-CoV-2 infection damages female reproductive health, causing significant reductions in AMH (−27.4%) and AFC (−1 antral follicle) values and an increase in FSH (+13.6%) and LH (+13.4%) values. No effect on E2 levels was reported. The pandemic has also affected the ability of infertile patients to access ART procedures, and that calls for a novel, updated blueprint designed to enhance our preparedness in the event that similar circumstances should occur again.

## 1. Introduction

Over the past four years, almost all of humanity has been overwhelmed by the SARS-CoV-2 pandemic, which has had significant repercussions in the health, economic, social, and political fields at unprecedented levels.

COVID-19 is classified as a respiratory disease that has dramatically changed our daily lives and can be transmitted primarily through respiratory droplets, however, there are recently published reports suggesting its ability to be transmitted through sexual intercourse, assisted reproductive technology, treatments, pregnancy, and breastfeeding.

The main clinical manifestations of SARS-CoV-2 infection range from asymptomatic to mild respiratory infection to severe disease, resulting not only in lung damage but also in multi-organ failure and death [1].

Currently available research findings show that other organs and systems can also be infected by the virus. In particular, investigations have been started to establish whether the reproductive system can be affected by the virus. Infertility can be defined as a disorder of the reproductive system that consists in the inability to achieve pregnancy after a period of at least 12 months of regular and unprotected sexual intercourse for women under 35 years of age, or at least 6 months for women over 35 years of age [2]. According to data from the World Health Organization (WHO), the problem of infertility affects approximately 15–20% of couples in advanced industrial countries [3,4].

The aim of this multicenter, observational, and prospective study is to evaluate how SARS-CoV-2 infection can directly and/or indirectly involve the female reproductive system, and, in particular, it highlights the possible consequences of the viral infection on the main indicators of female reproductive function that are an expression of the woman’s fertile potential: FSH, LH, estradiol (E2), AMH, and antral follicle count (AFC).

## 2. Materials and Methods

This is a multicenter, prospective, and observational study involving the reproductive medicine centers of the Ospedali Riuniti Villa Sofia-Cervello Hospital in Palermo and the Vanvitelli University. From September 2022 to March 2024, approximately 200 patients aged between 24 and 43 years with a diagnosis of infertility and a history of SARS-CoV-2 infection were enrolled. Symptomatic women, HIV-positive or other liver viruses, and patients with a history of ovarian cancer or who had undergone treatment with gonadotoxic drugs were excluded. Plasma measurements of FSH, LH, estradiol, AMH, and antral follicular count were performed before and after the infection. For the purpose of AMH measurement and for FSH, LH, and estradiol levels, patients were referred to laboratories relying on Roche Elecsys immunoassays (Roche Diagnostics, Basel, Switzerland), which is why we could safely assume the values to be dependable.

Given the retrospective nature of this study, post-COVID infection hormone levels were measured in patients in whom a confirmed COVID infection had been found, but with no need for hospitalization.

Also, in light of the slow and gradual resumption of medically assisted procreation activities, which were severely impacted by the pandemic, post-infection hormonal assessments could date back from 3 months up to about a year.

No patient was excluded at this point in time, since those who were not eligible for not meeting the criteria had already been automatically excluded from the study. Antral follicular count (AFC) was performed by transvaginal ultrasound through the GE Healthcare Voluson S10 3D/4D (GE Healthcare, Chicago, IL, USA) with 3D/4D probes either by Dr. Gullo (Principal Investigator of this study) or by his close collaborators, all properly trained and consistent in the procedure’s execution, which involved all patients who had already started a MAP pathway and who, as mentioned earlier, contracted COVID-19 with no need for hospitalization and no ongoing respiratory symptoms at the time of the examination. Endometriosis was diagnosed through the evaluation of the blood marker Ca125 and transvaginal ultrasound that showed the presence of typical ground glass images in correspondence with the ovaries and/or the presence of adenomyosis visualized by ultrasound. As it is well known, endometriosis is conclusively diagnosed via histological examination. This study has been registered on ClinicalTrials.gov with code NCT05435430 and has also been approved by the Palermo2 ethics committee, registry number 129 AOR 2022.

## 3. Results

The analysis accounting for the concentration of anti-Müllerian hormone (AMH) before and after COVID-19 infection showed that average concentration decreased from 1.33 ng/mL before SARS-CoV-2 infection to 0.97 ng/mL after infection. The average decrease following infection was −27.4% (95% CI: from −19.9% to −34.3%); this reduction is statistically significant at the 5% level (*p* < 0.0001).

The follicle count showed an average reduction of 1 follicle (95% CI: from −0.74 to −1.33), following SARS-CoV-2 infection. This reduction expressed in percentage terms is equal to −12.7% (CI 95%: from −9.1% to −16.3%) and is significant at the 5% level (*p* < 0.0001).

However, no significant variation was observed in the levels of E2 before and after SARS-CoV-2 infection.

Finally, a statistically significant increase in the average levels of FSH and LH emerged before and after SARS-CoV-2 infection.

In particular, FSH went from 7.7 to 8.8 mIU/mL, with an average increase of 13.6% (CI 95%: from 7.8% to 19.8%).

As regards LH, the mean values rose from 5.5 to 6.3 mIU/mL, with an average increase of 13.4% (95% CI: from 7.6% to 19.6%). A comparison of several significant values before and after SARS-CoV-2 infection in a sample of 203 women is laid out in Table 1.

Furthermore, some women’s conditions were considered as potentially able to modify the effect of SARS-CoV-2 infection on fertility, as considered in terms of reduction in AMH blood levels after COVID-19 compared to pre-existing levels.

**Figure 1:**
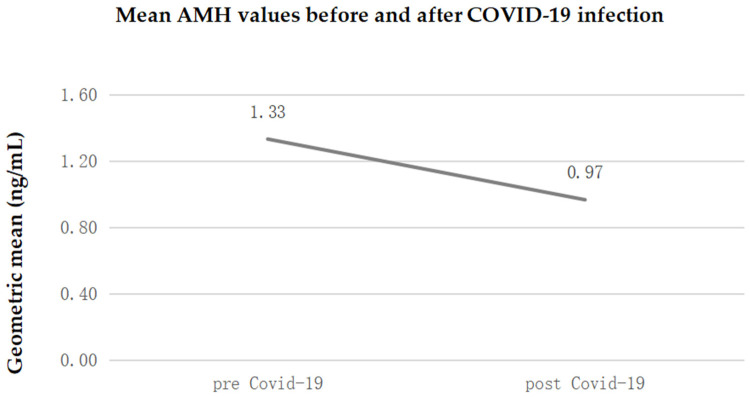
Mean AMH values before and after COVID-19 infection.

Table 2 shows how the overweight-obesity condition of women is able to accentuate the effects of the infection on the reduction in fertility; in particular, as Figure 1 shows, the ratio of the geometric means of AMH post/pre-COVID-19 was equal to 0.80 (i.e., −20%) among women with BMI ≤ 25 and 0.61 (i.e., −39%) among those with BMI > 25.

This means that in women with BMI ≤ 25, the average decrease in AMH levels was 20%, while in women with BMI > 25, the average decrease was 39% (compared to 27% if the entire sample is considered without taking into account the differences in BMI). This inhomogeneity between the two BMI classes is statistically significant at the 5% level.

Similarly, endometriosis exacerbates the effects of COVID-19: the ratio between the geometric means of AMH post/pre-COVID-19 was 0.76 (i.e., −24%) in the absence of endometriosis and 0.51 (i.e., −49%) in the presence of endometriosis. This difference is statistically significant at the 5% level.

On the other hand, age and pre-existing infertility do not modify the effects of COVID-19 in terms of reduction in AMH levels.

### Female Reproductive Function

Women’s reproductive function is guaranteed by the efficiency of the hypothalamus—pituitary—gonad axis. The hypothalamus is responsible for the production of important neurotransmitters involved in reproductive function. The anterior pituitary secretes its hormones under hypothalamic control.

In the hypothalamus, TRH, GnRH, GHRH, and CRH are produced.

Although the direct function on reproduction is linked to GnRH, other anomalies regarding the thyroid axis, adrenal axis, or anomalies of GH have important consequences on the reproductive mechanism.

The hormonal control mechanisms involve both the control of the long-circuit feedback (to the hypothalamus) and local control (at the ovarian level) [5].

Female fertility is the reproductive potential of a woman, that is, the possibility of having a child by having unprotected sexual intercourse. Menstruation can be defined as the marker of a woman’s reproductive life; it begins at 12 years of age with menarche, continues for a certain period of life interspersed with pregnancy and breastfeeding, and then ceases completely with menopause. For this to happen, the proper functioning of the hypothalamic–pituitary axis plays a crucial role.

At the hypothalamic level, GnRH is secreted, which acts on the pituitary gland, which in turn releases FSH/LH that allow, at the ovarian level, the secretion of estrogens, progesterone, androgens, inhibin A and B, and AMH. These are the key hormones of a woman’s reproductive life and regulate the endometrial cycle and the ovarian cycle.

At each menstrual cycle, a woman prepares for a pregnancy and does so through the maturation of a dominant follicle at the ovarian level and the maturation of the endometrium at the uterine level.

When pregnancy does not occur, luteolysis is activated and inflammatory mechanisms are triggered in which neutrophils, macrophages, and NK cells take part, with a significant release of cytokines and coagulation factors that allow uterine contractility and menstruation itself [6].

The fertile period is characterized by dynamics that both allow oocyte maturation and prepare the endometrium for possible embryo implantation.

This mechanism occurs cyclically due to some events mediated by the regulation of the hypothalamus–pituitary–ovary/endometrial axis through the pulsatile secretion of GnRH by the hypothalamus, the consequent release of gonadotropins by the pituitary (FSH, LH, and PRL), and ovarian steroidogenesis [7].

**Figure 2:**
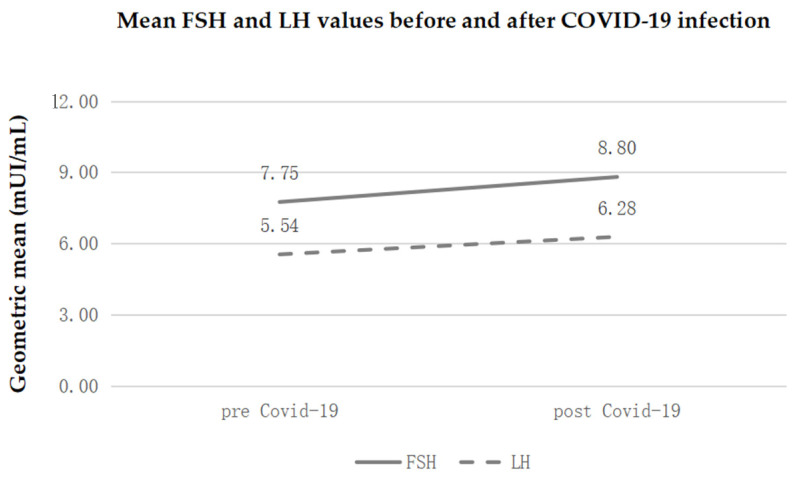
Mean FSH and LH values before and after COVID-19 infection.

In summary, during the first days of menstruation, the pituitary gland begins to produce increasing amounts of FSH, a hormone that stimulates the recruitment of ovarian follicles. Figure 2 shows mean FSH and LH values before and after COVID-19 infection.

**Figure 3:**
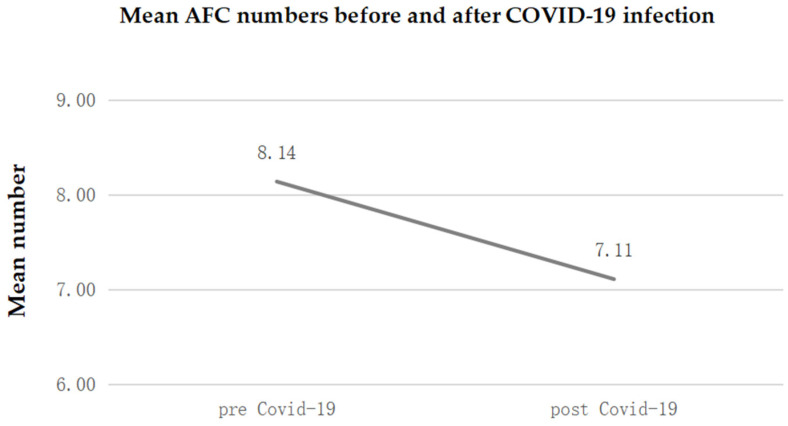
Mean AFC numbers before and after COVID-19 infection.

The growing follicles produce an increase in blood levels of estrogens that act on the uterus, determining the end of menstruation and the growth of the thickness of the endometrium for the purpose of better embryo implantation. With the selection of the dominant follicle, the production of estrogens continues to increase, determining the release of LH from the pituitary gland until its peak, which determines ovulation [8]. Figure 3 shows mean AFC numbers before and after COVID-19 infection.

After the follicle bursts, the corpus luteum forms on the ovary, which produces estrogens but above all progesterone, the latter essential for the maturation of the endometrium, which thus becomes definitively ready to welcome the possible embryo. In the absence of pregnancy, however, the corpus luteum degenerates after two weeks, and the consequent reduced ovarian production of estrogens and progesterone determines menstruation, that is, the shedding of the endometrium, with the start of a new cycle [9].

## 4. Discussion

We collected 203 patients diagnosed with infertility and a history of SARS-CoV-2 infection. We measured their FSH, LH, estradiol, AMH, and antral follicular count before and after infection. This study shows SARS-CoV-2 infection to have negatively affected female reproductive health, pointing to a statistically significant reduction in AMH (linked to ovarian response) and AFC values and a statistically significant increase in FSH and LH, although no effect on E2 levels was observed.

COVID-19 has been observed to potentially impact ovarian function, including the levels of AMH, which is a key marker of ovarian reserve [10]. Several studies suggest that women infected with COVID-19 may experience transient changes in AMH levels, likely due to the virus’s effects on the immune and endocrine systems [11,12]. The inflammation and immune response triggered by COVID-19 could influence ovarian function, leading to decreased AMH levels, which could indicate a temporary reduction in ovarian reserve [11]. However, long-term studies are still needed to fully understand whether these changes are reversible or if they have lasting effects on fertility. Additionally, factors such as disease severity, age, and underlying health conditions can also affect how COVID-19 influences AMH levels [13]. Our study also found a reduced AMH in females after COVID-19 infection.

Research data point to the ability of SARS-CoV-2 to determine a suppression of the hypothalamus–pituitary–gonad axis, with alteration of the normal cycle of FSH, LH, PRL, and estradiol [14]. There is also indirect evidence that SARS-CoV-2 could influence female fertility by directly involving the ovarian tissue and granulosa cells, thus decreasing oocyte quality and ovarian function. In a single-center observational study conducted in Wuhan, including 78 women of reproductive age, ovarian damage with a reduced ovarian reserve was observed in particular in patients affected by COVID-19 [15]. In some isolated cases, long-term sequelae of COVID-19 infection affecting female fertility have also been described [16]. Our study was comparable to previous studies, where elevated FSH and LH were observed in females after COVID-19 infection.

COVID-19 may also affect ovarian health by influencing AFC, which is a direct measure of ovarian reserve and fertility potential [17]. The antral follicles are small, early-stage follicles in the ovaries, and their number reflects a woman’s reproductive capacity [18]. Some research suggests that the systemic inflammation and immune response triggered by COVID-19 could disrupt ovarian function, leading to reduced AFC [15,19]. The virus’s ability to bind to ACE2 receptors, which are present in the ovaries, may also contribute to follicular dysfunction or loss [20,21]. These effects could be more pronounced in women who experience severe illness, as higher levels of inflammation could potentially impair folliculogenesis [22]. However, the exact mechanisms remain unclear, and further studies are needed to determine the long-term impact of COVID-19 on AFC and fertility outcomes, especially considering factors such as disease severity, age, and pre-existing reproductive conditions. Our study also found a reduced AFC in females after COVID-19 infection.

The study’s strength lies in its novelty and contribution to a rather underresearched area as of this writing. The study’s main weakness is the lack of a control group made up of patients without COVID-19 infection to draw relevant comparisons.

In addition to the clinical impact of the pandemic on female reproductive capabilities, it is worth noting that the pandemic severely affected ART provision due to national healthcare systems being overwhelmed by the emergency and restrictions thereof. As far as medically assisted reproduction is concerned, the COVID-19 pandemic has undoubtedly exacted a major toll on patients dealing with infertility conditions and seeking to achieve parenthood through ART. Italian data from the High Institute of Health (ISS) are quite telling in that respect: 9289 fewer cycles in the first four months of 2020 compared to 2019 in November 2020 were reported, 1500 fewer children born, and a steep reduction (34.1%) in ART activities, reaching as high as 40% in the country’s northern regions, which had been most severely impacted by the pandemic [23,24,25]. Such data, which appear consistent throughout Europe [26,27], should be viewed as a red flag in terms of highlighting the shortcomings of ART services provision, which national systems must improve and update, so that they can function in the event that another pandemic should strike in the future [26,27,28,29], requiring healthcare resources to be reallocated because of system oversaturation. Such updates must include the enactment of targeted legislative and regulatory measures [30] to make sure that prospective parents are not deprived of the opportunity to bring their ART pathway to fruition. A balanced, widely shared policy-making and legislative approach should also be framed when regulating access to innovative ART techniques [31,32,33,34,35], which may entail social and ethical challenges and complexities, which are likely to make proper regulation harder to frame due to their controversial and divisive nature [36,37,38,39,40,41]. The preservation of ART services during a crisis such as the one we have gone through is all the more essential in light of the recognition by the World Health Organization (WHO) that infertility is a disease [42] with the potential to negatively affect the psychosocial well-being of infertile couples, as also stressed by other major scientific societies and institutions such as the European Society for Human Reproduction and Embryology (ESHRE) [27], the American Society for Reproductive Medicine [43], and the International Federation of Fertility Societies in the guidelines issued in May 2020 [44]. Most patients going through the process of undergoing ART may be psychologically and emotionally vulnerable as well, anxious and afraid of the prospect of seeing their parenthood aspirations fail. Undoubtedly, delays and discontinuation of ART treatment can negatively affect the chances of success for such couples, thus doing irreparable damage to their family planning, also in light of the fact that age limits are often set as factors regulating access to ART in many countries [45,46,47]. Preventing such an outcome through evidence-based guidelines, updated adequately to meet the needs and challenges of extraordinary circumstances, is essential from an ethical as well as medicolegal perspective, as is the case with other obstetrical complications, which may impair reproductive capabilities [48,49,50,51]. The decision on the part of infertile couples to resort to assisted reproduction techniques in order to start a family of their own needs to be viewed as part and parcel of the right to health established by Article 32 of the Italian Constitution as well as the founding charter of the WHO, issued in 1946, which enshrine the right to a broad and comprehensive notion of health, not limited to the absence of disease, but rather as “a state of complete physical, mental and social well-being and not merely the absence of disease or infirmity” [52,53]. Such precepts make it a matter of essential human rights to be able to access ART services provided that there are the legal conditions to gain such access, in addition to the obligation for health administrations to make the availability of the aforementioned techniques possible through proper legal, regulatory, and organizational frameworks.

## 5. Conclusions

The data obtained show that SARS-CoV-2 infection seems to have negatively affected female reproductive health, causing a statistically significant reduction in AMH (−27.4%) and AFC (−1 antral follicle) values and a statistically significant increase in FSH (+13.6%) and LH (+13.4%), although no effect on E2 levels was observed.

It was also observed that some variables seem to be able to modify the effect of the virus on female fertility.

In particular, BMI seems to have a role in the consequences of COVID-19 in terms of AMH reduction, since women with BMI > 25 seem to be more affected by this effect (−39%); even in women with BMI < 25, a decrease in AMH levels was observed, but to a lesser extent (−20%).

Similarly, the condition of endometriosis also seems to be able to accentuate the effect of the virus (AMH −24% in the absence of endometriosis and −49% in the presence of endometriosis).

On the contrary, however, age and the presence or absence of infertility pre-existing to the infection do not seem to modify the effect of the virus.

It would be appropriate to continue with the evaluation of these data in order to evaluate the effect of the SARS-CoV-2 virus in the long term and to be able to clarify whether the consequences on female fertility are only transitory (and for how long) or permanent. Our working group also intends to continue the study by recruiting a control group of non-infected patients in order to evaluate the possible role of other variables other than COVID-19, but existing in the same period (such as stress and lockdown), on the reproductive potential of women. It is of utmost importance to continue our research efforts in order to deepen our understanding of the impact of COVID-19 on human reproductive capacity and consequently improve our global understanding of the strategies that can be implemented to eliminate potential sequelae. Just as importantly, the need for enhanced preparedness at the policy-/law-making and regulatory levels will be essential to make sure that the inalienable right to exercise one’s reproductive freedom is not adversely affected in future similar emergencies.

## Figures and Tables

**Table 1:** Fertility analysis: comparison between values before and after SARS-CoV-2 infection in a sample of 203 women.

Fertility Indicators	Pre-COVID-19 Average Values	Post-COVID-19 Average Values	Post/Pre-COVID-19 Relation	Variation % Post-Pre [CI 95%]		*p*-Value
AMH *	1.33	0.97	0.73	−27.4%	[−19.9%; −34.3%]	<0.001
AFC **	8.14	7.11	0.87	−12.7%	[−9.1%; −16.3%]	<0.001
Estradiol *	52.7	55.43	1.05	5.1%	[−3.3%; 14.3%]	n.s.
FSH *	7.75	8.80	1.14	13.6%	[7.8%; 19.8%]	<0.001
LH *	5.54	6.28	1.13	13.4%	[7.6%; 19.6%]	<0.001

[*] Values correspond to the geometric mean of the blood concentration. [**] Values correspond to the arithmetic mean of the follicular count.

**Table 2:** Analysis of the interaction between the woman’s demographic and clinical conditions and SARS-CoV-2 infection in terms of impact on fertility (assessed as variation in AMH hormone levels).

Patient Conditions		No.	AMH Levels Post-Pre-COVID-19 (Geometric Mean Ratio)	Relationship between Strata Values	Adjusted Ratio *	*p*-Value
Age						
	≤35 years	80	0.75	rif.	rif.	-
	>35 years	123	0.71	0.95	0.92	n.s.
BMI						
	≤25	128	0.80	rif.	rif.	-
	>25	75	0.61	0.76	0.76	<0.01
Infertility conditions						
	None	66	0.70	rif.	rif.	-
	Poor ovarian reserve	68	0.72	1.03	1.08	n.s.
	Other infertility conditions	69	0.76	1.09	1.10	n.s.
Endometriosis						
	Absent	182	0.76	rif.	rif.	-
	Present	21	0.51	0.67	0.64	<0.01

[*] Ratios between strata values adjusted for all conditions reported in the table using a log-linear regression model.

## Data Availability

All data are available upon request from the corresponding author.
